# Immunomodulatory Mechanisms of Mesenchymal Stem Cells and Their Potential Clinical Applications

**DOI:** 10.3390/ijms231710023

**Published:** 2022-09-02

**Authors:** Yutong Huang, Qiang Wu, Paul Kwong Hang Tam

**Affiliations:** 1 The State Key Laboratory of Quality Research in Chinese Medicine, Macau University of Science and Technology, Macau 999078, China; 2 Faculty of Medicine, Macau University of Science and Technology, Macau 999078, China

**Keywords:** mesenchymal stem cells (MSCs), immunomodulation, engineering, clinical applications

## Abstract

Mesenchymal stem cells (MSCs) are multipotent stem cells with the capacity of self-renewal, homing, and low immunogenicity. These distinct biological characteristics have already shown immense potential in regenerative medicine. MSCs also possess immunomodulatory properties that can maintain immune homeostasis when the immune response is over-activated or under-activated. The secretome of MSCs consists of cytokines, chemokines, signaling molecules, and growth factors, which effectively contribute to the regulation of immune and inflammatory responses. The immunomodulatory effects of MSCs can also be achieved through direct cell contact with microenvironmental factors and immune cells. Furthermore, preconditioned and engineered MSCs can specifically improve the immunomodulation effects in diverse clinical applications. These multifunctional properties of MSCs enable them to be used as a prospective therapeutic strategy to treat immune disorders, including autoimmune diseases and incurable inflammatory diseases. Here we review the recent exploration of immunomodulatory mechanisms of MSCs and briefly discuss the promotion of the genetically engineered MSCs. Additionally, we review the potential clinical applications of MSC-mediated immunomodulation in four types of immune diseases, including systemic lupus erythematosus, Crohn’s disease, graft-versus-host disease, and COVID-19.

## 1. Introduction

In the mid-twentieth century, researchers isolated a long-lived stromal cell from guinea pig bone marrow and found that these stromal cells can differentiate into various mesodermal cells, such as cardiomyocytes, adipocytes, and chondrocytes. Arnold Caplan believed that such stromal cells were similar to stem cells and possessed the same biological properties of multi-directional differentiation and self-renewal, and named these cells mesenchymal stem cells, with their abbreviation, MSCs, widely recognized by the biomedical and commercial communities [1]. In order to accurately define MSCs, the International Society for Cellular Therapy (ISCT) proposed their minimum identification criteria. Firstly, MSCs should grow adherently under standard culture conditions; secondly, MSCs must express CD73, CD90, and CD105 cell surface markers, but not the hematopoietic markers CD14, CD34, CD45, CD11b, CD79α, or the histocompatibility complex molecule HLA-DR; finally, MSCs should be able to be induced into chondrocytes, osteoblasts, and adipocytes [2]. MSCs can be derived from bone marrow, umbilical cord, and many other tissues. MSCs isolated from various tissues exhibit diverse cell surface markers that can be used in different therapeutic options.

MSCs possess the multi-directional differentiation potential and self-renewal ability, implying that these cells can differentiate into multiple cellular tissues and organs under appropriate circumstances. Additionally, MSCs also show low immunogenicity, a homing effect, and an immunomodulatory function. Based on their multifunctional characteristics, MSCs are very promising in the therapy of immune-related and inflammatory diseases. Especially in recent years, numerous studies have shown that MSCs have made new breakthrough in the treatment of intractable diseases. The immunomodulatory mechanisms of MSCs are primarily mediated by direct cell contact with immune cells and paracrine activity [3]. In the immune system, MSCs mainly exert immunosuppressive effects, thereby preventing an excessive immune response and modulating both innate and adaptive immune cells. In addition, MSC-derived exosomes and engineered MSCs could also enhance their biological characteristics to a certain degree, especially the immunomodulatory functions.

Additionally, the statistical results of ClinicalTrials.gov have shown that MSCs are a common and effective cell source in clinical cell-based therapy, and their clinical application accounts for approximately 7% of the clinical treatment of stem cells. Existing studies have proven that MSCs could be considered as a promising therapeutic option for the treatment of refractory inflammatory diseases, immune-related disorders, and even the novel coronavirus disease 2019 (COVID-19) due to their immunomodulatory effects [4,5]. However, prior to wide clinical applications of MSCs, a comprehensive understanding of the underlying mechanism is essential and crucial. In this review, we summarize the recent exploration of immunomodulatory effects of MSCs and discuss their potential clinical applications.

## 2. Immunomodulatory Effects of MSCs

The immune system mainly exerts immune surveillance, immune defense, and immune regulation functions, maintaining our body homeostasis. Immune dysfunction, such as insufficient or excessive immune responses, will cause corresponding diseases. Immunomodulation of MSCs involves the interactions with innate and adaptive immunity, mainly through direct cell contact with immune cells and the secretome of paracrine activity such as cytokines, chemokines, growth factors, and other impact factors.

### 2.1. Interaction of MSCs with Innate Immune Cells

Innate immunity is our body’s first two lines of defense against disease, which creates the corresponding immune responses to the invasion of pathogens. MSCs can modulate the immune response and promote the repair of inflammatory damage by interacting with innate immune cells [6].

#### 2.1.1. Natural Killer Cells

Activated natural killer cells (NK cells) can release cytotoxic factor (NKCF), perforin, and other pro-inflammatory cytokines while exerting cytotoxic effects [7]. A high proportion of MSCs can inhibit the proliferation, differentiation, and activation of NK cells [8,9].

MSCs can suppress the proliferation, interfere with the cytotoxicity, and inhibit the secretion of pro-inflammatory cytokines of NK cells [10]. The cytotoxicity of NK cells can be enhanced after activation by IL-2 or IL-15. When MSCs were co-cultured with NK cells, IL-2/IL-15, IFN-γ were downregulated and the growth of the NK cells was inhibited. In addition, the inhibition of the NK cells’ cytotoxicity was associated with a reduction in activating receptors such as NKG2D, NKp30, and NKp44 by MSCs. The secreted cytokines, such as PGE2, TNF-α, IDO, and HLA-G5, by MSCs play major inhibitory roles. The isoforms of HLA-G can be recognized by inhibitory receptors on the surface of NK cells, thereby inhibiting the secretion of IFN-γ and the cytotoxicity of NK cells [11].

MSCs cannot be lysed by quiescent NK cells in vivo, but activated NK cells can induce the death of MSCs [12,13]. MSCs modulate the activities of NK cells through activating receptor signaling rather than attenuating NK cell inhibitory signaling. In addition, after preconditioning by IFN-γ, the expression of activating signal ULBP3 can be downregulated while the inhibitory signal of MHC class I molecule expression can be upregulated, thereby protecting the MSCs from being lysed by the mass generation of IFN-γ and the activated NK cells under the inflammatory microenvironment. Hence, IFN-γ pretreatment can improve the tolerance of MSCs to the cytotoxic effect of NK cells [14].

#### 2.1.2. Macrophages

The differentiation and phenotype polarization of macrophages can be affected by their interaction with MSCs, which can secrete PGE2, indoleamine 2,3-dioxygenase (IDO), IL-10, and TGF-β [15].

After being stimulated by activation signals, macrophages can differentiate into the M1 phenotype while highly expressing pro-inflammatory factors, including TNF-α and IL-1β, subsequently playing the role in phagocytosing dead cells and invading pathogens. Macrophages in the M2 phenotype can secrete cytokines such as IL-10, which can control the pro-inflammatory response and thus strengthen the elimination of apoptotic cells, thus exerting an anti-inflammatory and damage-repair role. MSCs not only favor M2 anti-inflammatory phenotype polarization, but also inhibit their differentiation into the M1 pro-inflammatory phenotype and transform the phenotype of macrophages to M2 from M1 [16]. In a murine model of incision injury, researchers found that MSCs can migrate to the site of injury and induce macrophages to differentiate into the M2 phenotype, which is involved in repairing incision injury [17].

MSCs affect the differentiation of macrophages primarily through secretion of soluble cytokines. This mechanism is mainly related to the activity of IDO and the binding between PGE2 and EP2/EP4 receptors on the surface of macrophages. Among them, cyclooxygenase 2 (COX2) and IDO expressed by MSCs can be upregulated by the stimulation of pro-inflammatory factors TNF-α and IFN-γ, thereby further promoting the activation of M2 macrophages. TNF-α could activate TNFR1 and TLFR4 on the surface of MSCs, thereby activating NF-κβ signaling [18]. Thus, MSCs express COX2 and synthesize PGE2 which binds to EP2 or EP4 receptors to promote the production of IL-10 by macrophages, achieving the therapeutic purpose of inflammatory outcome and reducing the mortality of mice. Through an experimental model in which lung injury was induced by endotoxin, Choi et al. observed that downregulation of TNF-α, CXCL2, and the production of IL-10 by alveolar macrophages was reversed after infusing MSCs into the damaged murine lungs. Furthermore, MSCs can secrete TNF-stimulated gene 6 (TSG-6) and inhibit the inflammatory response. In addition, activated macrophages can secret TNF-α and induce MSCs to release TSG-6 which further directly interacts with CD44 expressed by macrophages to inhibit the negative feedback of NF-κβ signaling, exerting an anti-inflammatory effect [19].

Additionally, exosomes are critical in intercellular communication through exchanging microRNAs or proteins between cells. Hence, MSC-derived exosomes have proven to be a promising tool for the therapy of inflammatory disorders owing to their safety and better chemical stability. Through a murine model of myocardial ischemia–reperfusion injury, Zhao et al. found that injecting MSC-derived exosomes could inhibit the inflammatory factors and favor the M2 phenotype polarization by inhibiting miR-182 that targets the TLR4 receptors, thus converting M1 macrophages into M2 macrophages [20].

#### 2.1.3. Dendritic Cells

Dendritic cells (DCs) are antigen-presenting cells that sense antigen material from the external environment and presents it on the cell surface to the T cells of the immune system. Activated DCS are important in adaptive immune responses since they stimulate the generation of cytotoxic T lymphocytes (CTL), while immature DCs gradually differentiate into mature DCs under the stimulation of inflammatory factors or pathogens [21].

MSCs can restrain the differentiation, maturation, and antigen-presenting capability of DCs, interfering with the production of pro-inflammatory factors. In addition, MSCs can suppress the development of immature DCs and hinder CD34-derived precursor cells to differentiate into epidermal DCs. Ramasamy et al. found that monocytes were blocked in the G0 phase after co-culturing with MSCs. Additionally, co-culture of mature DCs with MSCs reduced the secretion of IL-12 and downregulated the expression of MHC I and MHC II molecules, cell surface molecule CD83, as well as the costimulatory molecules, thereby interfering with the antigen-presenting ability of DCs [22].

The mechanism of interaction between MSCs and DCs is similar to the aforementioned that MSCs favor the M2 phenotype polarization. MSCs can also transform DCs into an anti-inflammatory and tolerant phenotype. DCs can down-regulate the production of pro-inflammatory cytokines under the action of MSCs. However, tolerant phenotype DCs induced by MSCs fail to activate CD4^+^ T cells and suppress delayed-type hypersensitivity in vivo. Paczesny S et al. [23] observed that DCs activated by MSCs can promote the production of Tregs, thereby regulating immune function and maintaining immune tolerance. In addition, in vivo studies have shown that kidney transplanted patients still survived low-dose immunosuppressive agents though MSCs were used to induce DCs. Chiesa et al. [24] found that MSCs induced tolerogenic DCs by activating AKT, affecting NF-κβ signaling without influencing IL-10 secretion. Liu et al. [25] showed that murine MSCs generated a new group of tolerant DCs through the activation of suppressor of cytokine signaling 3 (SOCS3). Taken together, these studies suggest that MSC-induced tolerant DCs could exert an immunosuppressive effect, However, the specific mechanism of MSC-induced tolerant DCs still requires further exploration.

### 2.2. Interaction of MSCs with Adaptive Immune Cells

Adaptive immunity includes two interacting mechanisms: cellular immunity and humoral immunity. It plays a specific immune-regulation role. MSCs have been proven to exert immunomodulatory effects on adaptive immunity, mainly through direct cell–cell contact and paracrine activity.

#### 2.2.1. T Cells

The immune response of T cells includes the induction phase, response phase, and effector phase, after antigen-presenting cells (APCs) process and the antigen-presenting stage. Finally, T-cell receptors (TCRs) and costimulatory molecules will be activated [26].

Interestingly, MSCs can suppress the proliferation and activation of CD4^+^ T cells and CD8^+^ T cells, except for inducing their apoptosis. Di Nicola et al. confirmed that T cell proliferation induced by phytohemagglutinin stimulation or mixed lymphocyte culture could be inhibited by MSCs, and the inhibitory effect was dose dependent [27]. When MSCs interact with T cells, the cell cycle regulator p27kip1 was highly expressed at the molecular level, and the expression of cyclin D2 inhibition led to the arrest of T cell proliferation in the G0/G1 phase of the cell cycle. However, when MSCs were withdrawn and exogenous IL-2 was added, the quiescent T cells were reactivated and mediated the immune response again [28]. Furthermore, MSCs inhibited the cytolysis of CD8^+^ T cells in the mixed lymphoid system at the beginning of the culture, but when adding the MSCs during the lysis phase, the blocking effect could not be achieved [29]. Hence, MSCs may inhibit the formation of cytotoxic T cells to a certain extent during the afferent phase of the allogeneic reaction, but MSCs cannot reactivate the inhibitory effect if CD8+ T cells are activated.

Tse et al. confirmed that direct cell contacts between human-derived MSCs and T cells would suppress the proliferation of allogeneic T cells via in vivo studies [30]. MSCs can strongly attach to T cells through their adhesion molecules, thereby affecting the proliferation, activation, and differentiation of T cells [31]. In addition, MSCs highly express some chemokines that are related to the interaction of T cells. Neutralizing CXCR3, the receptor for these chemokines, can reverse the immunosuppressive effect of MSCs [32]. Ren et al. also found that the adhesion molecules ICAM-1 and VCAM-1, which are highly expressed in MSCs, were closely related to the interaction mechanism of regulatory T cells (Treg) [33]. These studies demonstrated that blocking these chemokines or adhesion molecules can significantly weaken the immunosuppressive effect mediated by MSCs. Up to date, more than 30 soluble cytokines secreted by MSCs have been shown to be involved in the immunomodulation of T cells, such as TGF-β, HGF, IDO, PGE2, IL-6, IL-10, NO, and Gal-9 [34].

Regulatory T cells (Treg cells) are primitive CD4^+^ T cells. Treg cells highly express Forkhead box P3 (Foxp3) and surface marker CD25 (IL-2 receptor), which exert immunomodulatory and anti-inflammatory effects [35]. Interestingly, MSCs can induce the aggregation and generation of Treg cells. MSCs expand Treg cells through an HLA-G5-dependent pathway, which is related to PGE2, TGF-β1 secreted by MSCs, and IL-10 secreted by Tr1 is critical for MSCs to express HLA-G5. In addition, CD4^+^ T cells can be induced to differentiate into Treg by activation of Notch-1 signaling [36]. By co-culturing MSCs and PBMCs, Melief et al. found that MSCs can induce monocytes to differentiate into M2 macrophages while IL-10 and CCL18 secreted by monocytes were indirectly involved in the generation of Treg cells [37]. Under the condition of co-culture, MSCs can transfer T cells from the canonical NF-κβ pathway to the non-canonical one, and this group of cells would highly express the immunosuppressive marker CD69 [38].

MSCs directly or indirectly affect the activation and differentiation of pro-inflammatory Th17 and Th1 cells [39]. PGE2 secreted by MSCs not only promotes the proliferation of Treg, but also blocks the differentiation of Th17 cells from naive T cells. Under an inflammatory microenvironment, MSCs directly adhere to Th17 cells through chemokine receptor 6 (CCR6) and transform these cells into Foxp3^+^ Treg cells [40]. Although MSCs can facilitate the generation of Treg cells, the in vivo experimental results are still controversial, and the specific immunomodulatory mechanism needs to be further explored.

#### 2.2.2. B Cells

The underlying mechanisms of how MSCs modulate B cells still remain elusive, but various in vitro experiments have confirmed that MSCs can suppress the proliferation, maturation, and antibody production of B cells to a certain extent. Glennie et al. found that the proliferation of B cells were blocked in the G0/G1 phase when MSCs were co-cultured with these B cells, though apoptosis of B cells were not induced [28]. In addition to blocking B cells proliferation, IL-1RA and CCL2 secreted by MSCs can inhibit the maturation and immunoglobulin antibodies production of B cells [41,42]. MSCs can also inhibit the expression of chemokine receptors located on the surface of B cells [43]. Further, MSCs can inhibit the proliferation of B cells and reduce antibody secretion via the PD-1/PD-L1 pathway [44].

Strikingly, the observation that inhibition of MSCs by B cells requires signals from T cells suggest that humoral immunity and cellular immunity interact with each other [45]. For instance, PGE2 and IDO, which are involved in cellular immunity, also exert immunosuppressive effect on B cell activity. Gerdoni et al. found that the antibody generation was inhibited and the response of PLP-specific T cells was also affected by MSCs [46]. Inoue et al. found that a single injection of MSCs with cyclophosphamide into patients with systemic lupus erythematosus could reduce autoantibodies against double-stranded DNA [47]. Moreover, T cells simultaneously can induce MSC-mediated suppression of B cell functions and Ig secretion [48]. It will be interesting to further interrogate the immunomodulatory mechanisms of MSCs in humoral immunity and cellular immunity.

### 2.3. Preconditioning of Pro-Inflammatory Factors

Pretreatment of MSCs with IFN-γ can improve the tolerance of MSCs under inflammatory stimuli, thereby decreasing the phagocytosis effect of cytotoxic cells and lymphocytes. IFN-γ has been proved to enhance the immunosuppressive effect through the IFN-γ-JAK-STAT1 pathway, and a low concentration of IFN-γ can stimulate the antigen presentation of MSCs, which can better survive in an inflammatory microenvironment [49].

In addition to IFN-γ, other pro-inflammatory factors also play pivotal roles in affecting the immune regulation of MSCs. TNF-α or IL-1α could induce IFN-γ-treated MSCs to express the necessary adhesion molecules, including ICAM-1 and VCAM-1, for mediating immunosuppressive effects. Besides, the simultaneous stimulation of activated MSCs with IFN-γ, IL-1α, and IL-1β can induce high NOS expression, which can catalyze the production of various leukocyte chemokines such as NO secretion. Groh et al. [50] found that IL-1β secreted by monocytes can stimulate MSCs to secrete TGF-β, strengthening the inhibition of T cells.

MSCs primed with pro-inflammatory factors can enhance the inhibition of the T cells proliferation [51]. In addition, the combination of TNF-α and IFN-γ can also promote the expression of some cytokines such as COX-2, HGF, and PGE2 in MSCs that are beneficial to impede the proliferation of T cells. The combined use of these pro-inflammatory factors also participates in the expression of chemokines such as CCR5, CCR10, CXCL9, thereby inhibiting the proliferation of immune effector cells [14].

### 2.4. Gene Engineered MSCs

Cell-based gene therapy has become a promising treatment option. Previously scientists utilized physical or chemical reactions and pharmaceuticals to precondition MSCs, aiming to improve their immunomodulation function. However, the effect of this conventional pre-treatment method is transient and instable. Genetic modification of targeted cells could promote the therapeutic effect to a certain degree [52]. Genetic engineered MSCs also exert more curative effect while comparing to non-genetically modified MSCs.

Mei et al. reported that MSCs overexpressing angiopoietin-1 can improve the therapeutic effect in mice with acute lung injury (ALI). Angiopoietin-1 possesses the ability to decrease endothelial permeability and hinder leukocyte-endothelial interactions, and hence high expression level of angiopoietin-1 may exhibit better treatment result [53]. Subsequently, Maria Florian et al. found that the angiopoietin-1 transgene which was efficiently and persistently expressed in MSCs could enhance the therapeutic effect in ALI mice models [54].

The self-renewal and low immunogenicity properties of MSCs seem to have broad applications in tissue transplantation, but the regenerative ability of the transplanted cells might be decreased due to the harsh environment caused by diseases, thereby affecting the clinical efficacy. Shu et al. [55] explored the therapeutic significance of various genes used in the gene engineered MSCs and found that the regenerative characteristics of MSCs could be ameliorated to a certain degree in the therapy of early-stage osteonecrosis of the femoral head after applying genetic engineering.

Taken together, these studies have proved that gene engineered MSCs possess the capacity of increasing their therapeutic effect via enhancing the immunomodulation and regenerative effect [56]. However, there are still many obstacles and challenges before widely applying this method can achieve wide clinical applications. For instance, the most appropriate delivery cell dose, the distribution in the body and long-term safety are issues that are still required to be adequately addressed.

## 3. Potential Clinical Applications of MSC-Mediated Immunomodulation

Hormone drugs or non-steroidal anti-inflammatory drugs are commonly applied to relieve the symptoms of refractory inflammatory diseases and autoimmune diseases. However, it’s highly possible for these drugs to cause unexpected side effects. In recent years, the great potential of MSC-based treatment has been shown in treating immune disorders due to their considerable immunomodulatory efficacy.

### 3.1. Systemic Lupus Erythematosus

Systemic lupus erythematosus (SLE) is an autoimmune disease caused by an excessive immune response, leading to tissue necrosis or organ inflammation. The functions of T and B cells from patients with SLE were disordered, and the expression level of pro-inflammatory cytokines was higher than that of healthy people [57]. Intriguingly, the biological activity and immunomodulatory function of MSCs which were isolated from SLE patients failed to modulate the immune system, indicating that SLE-MSCs exhibited obvious difference from normal MSCs [58,59].

MSCs possess the capability of damage repair, anti-inflammatory properties and immunomodulation. Indeed, bone marrow MSCs (BM-MSCs) and dental tissue-derived MSCs transplantation into SLE murine models exerted obvious therapeutic effect [60]. Consistently, some recent clinical trials have also proved the safety and therapeutic effect of MSCs in patients with SLE. For example, a phase I clinical trial (Clinical Trial Number: NCT03171194) conducted between April 2017 and October 2018 on 6 adult participants with active SLE demonstrated the safety and efficacy of umbilical cord MSCs. After the infusion of MSCs incremental transitional B cells as well as reduced activated naïve B cells were detected in 5 patients, increased Helios^+^ Treg cells were found in 2 patients, GARP-TGFβ remarkably increased in all patients [61]. Another recent phase I clinical trial (Clinical Trial Number: NCT03174587) conducted between May 2017 and November 2019 confirmed that MSCs were safe and could relieve the inflammatory symptoms to a certain degree and decreased generation of autoantibodies and regulation of immune cell activity were observed in this trial [62]. These studies have shown the safety and considerable therapeutic effect of MSCs. However, more relevant clinical trials are still required to clarify the detailed MSCs-mediated immunomodulatory mechanisms before widely applying MSCs-based therapy to treat this refractory autoimmune disease.

### 3.2. Crohn’s Disease

Crohn’s disease (CD) is a type of inflammatory bowel disease that mostly affects the terminal ileum and right colon, leading to abdominal pain, intestinal obstruction, diarrhea and other digestive disorders [63]. CD is distinguished by severe colonic inflammation induced by Th1 cells and associated with a high expression level of proinflammatory cytokines [64].

MSC administration could relieve insufferable symptoms, thereby improving the life quality of patients with CD. Injection of human-derived MSCs exhibited significant therapeutic effect in CD animal models via reducing Th1-mediated inflammatory responses and ameliorating the activity of Treg cells population [65,66]. Further, human MSCs infusion can reduce the expression level of associated proinflammatory cytokines IL-2 and IL-6 [67]. In addition, MSC injection can also relieve the symptoms of abdominal pain, weight loss and intestinal inflammation, thereby increasing the percentage of survival in CD subjects [65].

In addition to in vitro and in vivo animal studies, recent completed clinical trials have provided a proof of concept that MSC-based therapy can relieve symptoms of CD. In an experimental investigational treatment with autologous BM-MSCs, 7 of 12 patients with CD achieved symptoms relief, indicating the feasibility of the MSCs-based therapy [68]. Gao et al. found that MSC administration can alleviate the severity of colitis by regulating the proliferation of intestinal epithelial cells and improving the immune function of T cells [69]. Similarly, a recent double-blind controlled phase III trial (Clinical Trial Number: NCT01541579) conducted on 212 CD patients evaluated the efficacy of MSC treatment s and reported that MSC-treated patients received better clinical remission and less adverse reactions [70]. An extended follow-up study of this trial demonstrated the long-term safety and well-tolerated of MSCs treatment [71]. Another phase I/II trial (Clinical Trial Number: NCT01144962) displayed the injection of allogenic bone marrow derived MSCs is a decent strategy to promote healing efficacy on CD patients and no related adverse reactions were found in this trial [72]. Moreover, a phase I study (Clinical Trial Number: NCT01915927) conducted on perianal CD patients indicated that the transfer of autologous MSCs into a bioabsorbable matrix reduced the length and diameter of the injured fistula, achieved clinical healing and radiographic marker response [73]. Taken together, these clinical trials suggested that application of appropriate autologous or allogenic MSCs is able to alleviate normal disease symptoms, thus contributing to the efficient treatment of chronic perianal CD.

### 3.3. Graft-versus-Host Disease

Graft-versus-host disease (GVHD) is a serious multisystem inflammatory disease caused by a cytotoxic attack by T cells in allogeneic donor grafts that target a recipient’s target cells after transplantation. The donor’s immune system’s white blood cells reject the recipient in this inflammatory response.

MSCs could be a decent option to treat GVHD due to their multipotent characteristics [74].

As mentioned above, genetically engineered MSCs could overexpress or block specific genes via genetic modification, thereby specifically enhance or modulate the immune response. MSCs overexpressing ICAM-1 can affect the functions of the DCs and T cells, hindering their maturation, differentiation, and proliferation. In a GVHD animal model, Li et al. found that the MSCs-ICAM-1 infusion significantly increased the survival of experimental subjects while increasing Treg cells proliferation and restraining DCs maturation [75]. Moreover, MSCs-CXCR4 administration can decrease proinflammatory cytokines associated with GVHD while enhancing anti-inflammatory cytokines in a murine GVHD model, thereby improving the survival rate of MSC-CXCR4 group [76].

In a phase II/III clinical trial, bone marrow MSCs (JR-031) administration obviously ameliorated the survival rate of the patients without side effects [77]. Similarly, a phase II trial (Clinical Trial Number: NCT00504803) demonstrated that infusion of MSCs reduced GVHD mortality without eliminating the graft-versus-tumor effect in patients with hematological malignancies [78]. Another phase II double-blind controlled study demonstrated that infusion of umbilical cord MSCs relieved chronic GVHD symptoms after HLA-haploidentical stem cell transplantation. This treatment can downregulate the number of NK cells, enhance Treg cell function, and cause Th2 subtype differentiation [79]. Interestingly, a phase I study (Clinical Trial Number: NCT02923375) verified the safety and efficacy of induced pluripotent stem cell-derived MSCs in treatment of acute GVHD [80]. Moreover, a recent phase III prospective trial (Clinical Trial Number: NCT02336230) conducted in 54 pediatric patients with acute GVHD provided a robust outcome that infusion of MSCs is a potential replacement strategy for the failed steroid therapy [81].

### 3.4. COVID-19 Pandemic

Coronavirus disease 2019 (COVID-19) is an infectious disease caused by the SARS-CoV-2 virus. People infected by the virus generally suffer from cough, fever, and fatigue, or even multiple organ damage (MOD) [82]. Patients who recover from this disease may still possess a series of sequelae [83].

After SARS-CoV-2 invaded the body, it will cause a strong inflammatory response, accompanied by abundant secretion of inflammatory mediators, thereby generating a cytokine storm. The plasma levels in patients with COVID-19 showed that IL-2, IL-6, IFN-γ, monocyte chemotactic peptide (MCP)-1, TNF-α, and other proinflammatory factors were apparently higher than that in healthy people, especially the patients who were admitted to ICU [84]. Notably, MSCs can reverse the cytokine storm and inhibit over-activation of the immune response due to their multipotent characteristics and immunomodulatory effects [85]. Stimulated by high levels of IL-6, MSCs can adaptively produce mirR-455-3p-enriched cytokines and exosomes, alleviating cytokine storm and treating acute inflammatory liver injury [86]. Besides, MSC infusion can protect alveolar epithelial cells and promote neovascularization, further preventing lung fibrosis. Keratinocyte growth factor (KGF) secreted by MSCs promote alveolar fluid clearance and alleviate endotoxin-induced acute lung injury by upregulating the α1 subunit of ACE-2 [87].

A MSC-based clinical trial to treat COVID-19 was conducted in Beijing Hospital in 2020. Upon completion of this study, augmentation in peripheral lymphocytes, reduction in C-reaction protein, and the disappearance of hyperactivated cytokine-secreting immune cells were observed, indicating the safety and efficacy of the MSC treatment [88]. Additionally, MSC exosomes reduced lung injury and inhibited the proliferation and activation of CD4^+^, CD8^+^ T cells, and NK cells [89]. Correspondingly, another trial using BM-MSC-derived exosomes (ExoFlo™) in severe COVID-19 patients demonstrated the safety and considerable treatment effect of this method, suggesting MSC exosomes could be a potential option for treating COVID-19 [90]. For example, a double-blind controlled phase II trial (Clinical Trial Number: NCT04288102) recruited severe COVID-19 patients with lung damage to be treated with infusion of MSCs. The ratio of solid component lesion volume remarkably decreased in the patients treated with umbilical cord MSCs without obvious side effects [91]. Additionally, a phase I/II double-blind trial (Clinical Trial Number: NCT04333368) conducted on 45 patients with COVID-19-associated acute respiratory distress syndrome (ARDS) indicated that the difference in PaO_2_/FiO_2_ changes between the UC-MSCs infusion group and placebo group was not obvious within one week of administration [92]. In addition to treating severe COVID-19 patients, a phase I double-blind trial (Clinical Trial Number: NCT04535856) conducted on 9 patients with low clinical risk COVID-19 infection demonstrated the safety of MSC infusion [93]. To date, 21 completed MSC-based clinical trials for COVID-19 have been registered in ClinicalTrials.gov (19 August 2022) and various impressive outcomes were observed. Taken together, MSCs can indeed alleviate the exacerbation of COVID-19 symptoms. Yet, further in vivo studies and clinical trials to clarify the detailed immunomodulatory mechanisms of MSCs in treating COVID-19 are needed.

## 4. Conclusions and Prospects

**Figure 1:**
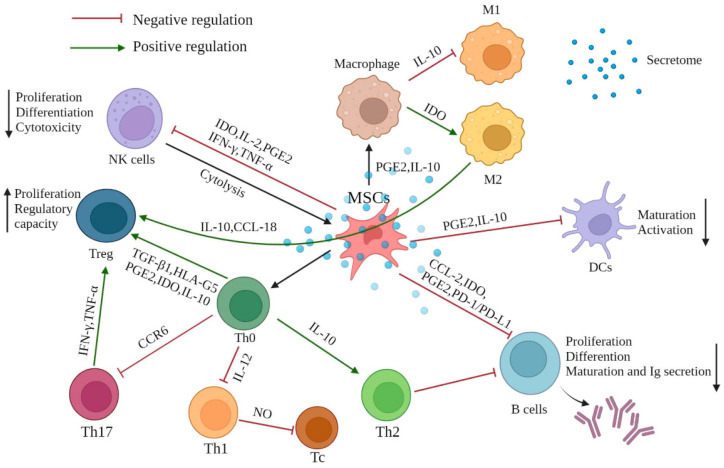
The effect of MSC-mediated immunomodulation on immune cells. MSCs exhibit immunomodulatory effects mainly through direct cell contact with innate and adaptive immune cells and the multifunctional secretome produced by the of paracrine mechanism of MSCs. MSCs mostly exert an immunosuppressive effect (red inhibitor), while performing positive regulation on generation of Treg cells, differentiation of Th2, and M2 macrophage polarization (green arrows). Various cytokines, chemokines, signaling molecules, and growth factors are involved in this mechanism, maintaining the immune homeostasis when the immune response is over-activated or under-activated. PGE2: prostaglandin E2; IDO: indoleamine-pyrrole 2,3-dioxygenase; IFN-γ: interferon-γ; TNF-α: tumor necrosis factor-α; TGF-β: transforming growth factor-β; IL: interleukin, PD-1/PD-L1: programmed death-1/programmed death-ligand 1; CCR6: chemokine receptor 6; CCL-2: C-C motif chemokine ligand 2; CCL-18: C-C motif chemokine ligand 18.

MSCs hold great potential in regenerative medicine and treatment of immune-mediated disorders because of their multipotent characteristics and immunomodulatory effects. MSCs exert this modulatory function mainly through direct interaction with immune cells and paracrine activity triggered by chemokines, cytokines, growth factors, and other inflammatory stimuli. MSC-derived exosomes could also be a promising treatment candidate since exosomes maintain the therapeutic value of the cells from which they are derived, with greater safety and without misgivings such as probable tumorigenesis and mutation of MSCs. Furthermore, preconditioning with proinflammatory cytokines and genetically modification through overexpressing or blocking specific genes in the MSCs could enhance the immunomodulation activity. Taken together, MSCs are generally effective, controllable, and feasible in treating immune diseases (Figure 1). Based on the progressive accumulation of preclinical and clinical mechanistic evidence, it can be expected that MSC-based therapy for the treatment of immune disorders will be further developed.

Though MSC-based therapy has gradually become mature, their prospective effects on clinical results have not yet been credibly achieved. There are still obstacles and challenges before widely applying MSCs for clinical use. It is noteworthy that MSCs are usually heterogeneous and vary widely under inflammatory or anti-inflammatory microenvironments, resulting in varied effects of MSC-mediated immunomodulatory functions. The impact of acute or chronic inflammation on MSC-mediated immunomodulation requires more in-depth exploration in the future. Besides, many factors, including microenvironments around the lesion sites, can affect the survival time and homing ability of the injected MSCs. More effort also should be put into optimizing the preconditioning methods to enhance the efficacy of MSCs and minimize the changes in MSC paracrine availability and therapeutic effects, especially in clinical transplantation.

The divergence between the outcomes of in vitro and in vivo studies should be further investigated. Liu H. et al. [94] found that MSC-derived osteogenic cells maintained their immunogenicity and immunomodulatory function in vitro. However, these functions were lost after osteogenic cells infusion. This suggests the importance of elucidation of MSC immunomodulation after switching from in vitro observation to in vivo studies. Though various completed clinical trials have been documented (Table 1), further clinical tests are still required. Moreover, MSCs derived from different tissues or organs may widely vary in gene expression, differentiation potential, and immunomodulatory activity. Therefore, it is also necessary to carefully choose the appropriate source of MSCs to better harness the clinical potential of the MSCs. Nevertheless, MSCs will be equipped with superior clinical value for the time to come if the detailed immunomodulatory mechanisms of MSCs can be elucidated and an increase in the number of preclinical and clinical studies can be achieved.

## Figures and Tables

**Table 1:** Completed clinical trials of MSC-based treatment for immune disorders.

Disease Type	Interventions	Number of Patients	Study Phase	NCT Number	Outcome	Ref.
SLE	UC-MSCs	6	Phase 1	NCT03171194	Increased GARP-TGFβ	[61]
SLE	Pooled allogenic olfactory mucosa-MSCs	7	Phase 1/2	NCT04184258	No results posted	Not provided
SLE	BM-MSCs	7	Phase 1	NCT03174587	Decreased generation of autoantibodies	[62]
CD	Autologous AT-MSCs	15	Phase 1/2	NCT01157650	Recovered external opening	[95]
CD	UC-MSCs	82	Phase 1/2	NCT02445547	No results posted	Not provided
CD	Allogenic BM-MSCs	21	Phase 1/2	NCT01144962	Perianal fistula healed gradually, no associated adverse events	[72]
CD	Cx601 AT-MSCs	278	Phase 3	NCT01541579	Long-term safety, well tolerated	[71]
CD	MSC-AFP	5	Phase 1	NCT03220243	No results posted	Not provided
CD	MSC-AFP	20	Phase1	NCT01915927	Decreased length and diameter of fistula tract	[73]
CD	PROCHYMAL^®^ adult human MSCs	98	Phase 3	NCT00543374	No results posted	Not provided
CD	PROCHYMAL^®^ adult human MSCs	330	Phase 3	NCT00482092	No results posted	Not provided
CD	PROCHYMAL^®^ adult human MSCs	73	Phase 3	NCT01233960	No results posted	Not provided
CD	PROCHYMAL^TM^ adult human MSCs	10	Phase 2	NCT00294112	No results posted	Not provided
GVHD	Allogenic MSCs	15	Phase 1/2	NCT01956903	No results posted	Not provided
GVHD	AT-MSCs	19	Phase 1/2	NCT01222039	No results posted	Not provided
GVHD	BM-MSCs	10	Phase 1/2	NCT02824653	No results posted	Not provided
GVHD	BM-MSCs	30	Phase 2	NCT00504803	Promoted engraftment and prevent GVHD	[78]
GVHD	PROCHYMAL^®^ adult human MSCs	260	Phase 3	NCT00366145	Well tolerated and no associated toxicities	[96]
GVHD	PROCHYMAL^®^ adult human MSCs	32	Phase 2	NCT00136903	No associated toxicities or ectopic tissue formations	[97]
GVHD	BM-MSCs	10	Phase 1	NCT01318330	No results posted	Not provided
GVHD	Mesenchymoangioblast-MSCs	16	Phase 1	NCT02923375	Safe, well tolerated and no serious adverse events	[80]
GVHD	PROCHYMAL^®^ adult human MSCs	11	Phase 2	NCT00284986	No results posted	Not provided
GVHD	MSC (hPPL)	50	Phase 1/2	NCT00827398	No results posted	Not provided
GVHD	UC-MSCs	10	Phase 1/2	NCT00823316	No results posted	Not provided
GVHD	PROCHYMAL^®^ adult human MSCs	192	Phase 3	NCT00562497	No results posted	Not provided
GVHD	MSCs infusion in Haplo-SCT	6	Phase 3	NCT03106662	No results posted	Not provided
GVHD	PROCHYMAL^®^ adult human MSCs	55	Phase 3	NCT02336230	Improved response rate and increased survival	[81]
COVID-19	MSCs secretome	40	Phase 3	NCT05122234	No results posted	Not provided
COVID-19	UC-MSCs	100	Phase 2	NCT04288102	Improved lung lesion volume, reduced solid component lesion volume	[91]
COVID-19	AT-MSCs	56	Phase 2	NCT04349631	No results posted	Not provided
COVID-19	AT-MSCs	55	Phase 2	NCT04348435	No results posted	Not provided
COVID-19	Wharton’s Jelly MSCs	30	Phase 2	NCT04625738	No results posted	Not provided
COVID-19	UC-MSCs	40	Phase 1	NCT04573270	No results posted	Not provided
COVID-19	Pooled allogenic olfactory mucosa-MSCs	32	Phase 1/2	NCT04382547	No results posted	Not provided
COVID-19	UC-MSCs + Heparin	24	Phase 1/2	NCT04355728	No results posted	Not provided
COVID-19	Allogenic MSCs	9	Phase 1	NCT04535856	No progression of severity	[93]
COVID-19	Allogenic AT-MSCs	26	Phase 1/2	NCT04366323	No results posted	Not provided
COVID-19	BM-MSCs extracellular vesicles	120	Phase 2	NCT04493242	Oxygenation restored, cytokine storm reduced and immunity modulated	[90]
COVID-19	Armed Forces BM-MSCs	600	Not applicable	NCT04492501	No results posted	Not provided
COVID-19	AT-MSCs	6	Phase 1	NCT04522986	No results posted	Not provided
COVID-19	UC-MSCs	30	Phase 1/2	NCT04392778	No results posted	Not provided
COVID-19	Allogenic MSCs	24	Phase 2	NCT04361942	No results posted	Not provided
COVID-19	MSCs exosomes	30	Phase 1/2	NCT04491240	No results posted	Not provided
COVID-19	UC-MSCs	15	Phase 1/2	NCT04400032	No results posted	Not provided
COVID-19	UC-MSCs	47	Phase 1/2	NCT04333368	No obvious difference in PaO_2_/FiO_2_ changes between UC-MSCs infusion group and placebo-treated group	[92]

UC: umbilical cord; BM: bone marrow; AT: adipose tissue; MSC-AFP: MSC-coated Gore Bio-A fistula plug; MSC (hppL): expansion of MSCs with human plasma and platelet lysate; MSC infusion in Haplo-SCT: MSC transfusion in haploid hematopoietic stem cell transplantation.

## Data Availability

This study did not report any data.
